# Effect of mind map-guided nursing on pain control after transurethral resection of the prostate

**DOI:** 10.1590/1414-431X2025e15233

**Published:** 2026-04-17

**Authors:** Nan Zhao, Eryun Tian

**Affiliations:** 1 Department of Urology, Third Medical Center, PLA General Hospital, Beijing, China; 2 Department of Organ Transplantation, Third Medical Center, PLA General Hospital, Beijing, China

**Keywords:** Transurethral resection of the prostate, Analgesic effect, Mind map, Nursing intervention, Pain management

## Abstract

We aimed to analyze the effect of mind map-guided nursing on pain control after transurethral resection of the prostate (TURP). Eighty patients with benign prostatic hyperplasia undergoing TURP were randomly separated into either a control group (routine perioperative nursing) or an intervention group (mind map-guided nursing). Visual Analog Scale (VAS) scores were employed to compare pain levels at 24 and 48 h postoperatively. Additional outcomes included the number of patient-controlled analgesia activations, time to first flatus, and length of hospital stay. Quality of life (Short Form-36 (SF-36)), sleep quality (Pittsburgh Sleep Quality Index (PSQI)), anxiety and depression (Self-Rating Anxiety Scale (SAS) and Self-Rating Depression Scale (SDS)), and prostate function (International Prostate Symptom Score (IPSS)) were evaluated before and after nursing interventions. Patient satisfaction with nursing care was also compared. After nursing interventions, the intervention group exhibited lower VAS scores at 24 and 48 h postoperatively, fewer analgesia pump activations, shorter time to first flatus, and reduced length of hospital stay, higher SF-36 scores across all dimensions, lower PSQI, SAS, SDS, and IPSS scores, as well as higher nursing satisfaction versus the control group (all P<0.05). Mind map-guided nursing alleviated postoperative pain following TURP, enhanced quality of life, sleep quality, and mental state, promoted prostate function recovery, and enhanced patient satisfaction with nursing.

## Introduction

Benign prostatic hyperplasia (BPH) is a histopathological condition characterized by nonmalignant proliferation of prostate tissue, often leading to benign prostatic enlargement and lower urinary tract symptoms (1). Since its introduction in 1943, transurethral resection of the prostate (TURP) has remained the gold-standard surgical treatment for bladder outlet obstruction due to BPH, owing to its established efficacy, cost-effectiveness, and central role in urological training (2-4). However, a number of postoperative complications, such as TURP syndrome, infections, and urethral or bladder neck strictures, may occur (5). Additionally, patients often experience pain after TURP, which not only affects their comfort, but may also impact their postoperative recovery and quality of life (6). Therefore, incorporating pain management as a component of postoperative care is essential for facilitating patient recovery.

The mind map is a visual representation tool employed across various medical education disciplines to organize and present ideas centered around a main concept, with related thoughts branching out into various subtopics or categories (7). It is organized in a radial and more lateral format, with the subject of study at the core and its specifics branching out towards the outskirts (7). Mind map integrates images and logical thinking through a combination of pictures and text, reflecting a natural process that aligns with the brain's divergent thinking patterns and aids patients in comprehending memory (8). The research conducted by Li et al. (8) indicates that mind map-based pain education in nursing care can significantly enhance daily functioning, alleviate pain symptoms, and enhance the quality of life of colorectal cancer patients post-surgery, offering valuable insights for their post-operative nursing care. Furthermore, studies have reported that implementing a predictive nursing program based on a mind map in patients undergoing high-concentration contrast three-dimensional computed tomography (CT) imaging of hepatic vessels improves CT scan quality, alleviates depression and anxiety, improves patient satisfaction with nursing guidance during inspection, and reduces the incidence of contrast medium leakage and iodinated contrast allergic reactions (8). Another study reveals that incorporating mind maps and behavior rating scales into the post-radiofrequency ablation care regimen boosts medication adherence, compliance with medical instructions, and the overall quality of life for atrial fibrillation patients (9). Given the proven benefits of mind map-based interventions in various clinical contexts, its application to postoperative pain control following TURP warrants investigation.

Taken together, a mind map, as a visual thinking tool, organizes complex information into a clear and easily understandable structure through elements such as graphics, lines, and colors. In the field of nursing, applying the mind map to postoperative pain control represents an innovative approach. Furthermore, there are few studies on the application of mind map-guided nursing in postoperative care following TURP, highlighting the novelty of this research. Hence, this study aimed to explore the effect of mind map-guided nursing on postoperative pain control after TURP.

## Material and Methods

### Ethical statement

This study was reviewed and approved by the Ethics Committee of the Third Medical Center, PLA General Hospital (China). All patients and their families were informed about the study and provided written informed consent prior to participation.

### Study participants

Eighty patients with BPH who underwent TURP at the Third Medical Center, PLA General Hospital, between January 2022 and December 2024 were enrolled. Inclusion criteria were: 1) patients meeting the diagnostic criteria for BPH and presenting with symptoms such as paroxysmal lower abdominal pain, bladder distension, urgency, suprapubic tenderness, microscopic or gross hematuria, and blood-tinged urine leaking around the catheter (10); 2) patients with a Mini-Mental State Examination score >24 and good communication skills; and 3) patients and their families who were informed about the study and provided consent to participate. Exclusion criteria were: 1) patients with other diseases causing urination obstruction, such as urethral stones, bladder stones, prostate cancer, or urethral stricture; 2) patients with severe dysfunction of heart, liver, kidneys, and other vital organs; 3) patients with malignant tumors, systemic immune diseases, severe infections, or other serious comorbidities; 4) patients with cognitive impairment or psychiatric disorders that prevented cooperation; and 5) patients with incomplete medical records.

Patients were randomly assigned into the intervention and control groups. The control group included 40 patients, with a mean age of 59.10±8.31 years and a disease duration of 8.90±2.46 years. The intervention group included 40 patients, with a mean age of 60.33±7.56 years and a disease duration of 8.78±2.56 years. No statistical differences were observed in general information between the two groups (P>0.05), indicating good comparability.

### Methods

The control group received routine perioperative nursing, including: 1) *Health education*: a series of structured health education activities were conducted, and detailed knowledge manuals were distributed to patients. The manuals introduced the diagnostic and therapeutic approaches for BPH, explained the risks of postoperative complications and their preventive strategies, and emphasized the importance of rehabilitation exercises and rational medication use for postoperative recovery. 2) *Postoperative nursing*: after surgery, the urinary catheter was indwelled and secured. Bladder irrigation was discontinued after 1 day, the characteristics of the drainage fluid were carefully monitored, and pain management as well as rehabilitation training were provided. 3) *Telephone follow-up*: following discharge, telephone follow-up was conducted every 2 weeks to assess the patient's recovery progress. 4) *Family support*: family members and close friends were encouraged to provide continuous encouragement and support, thereby reinforcing the patients' confidence in overcoming the disease.

**Figure 1:**
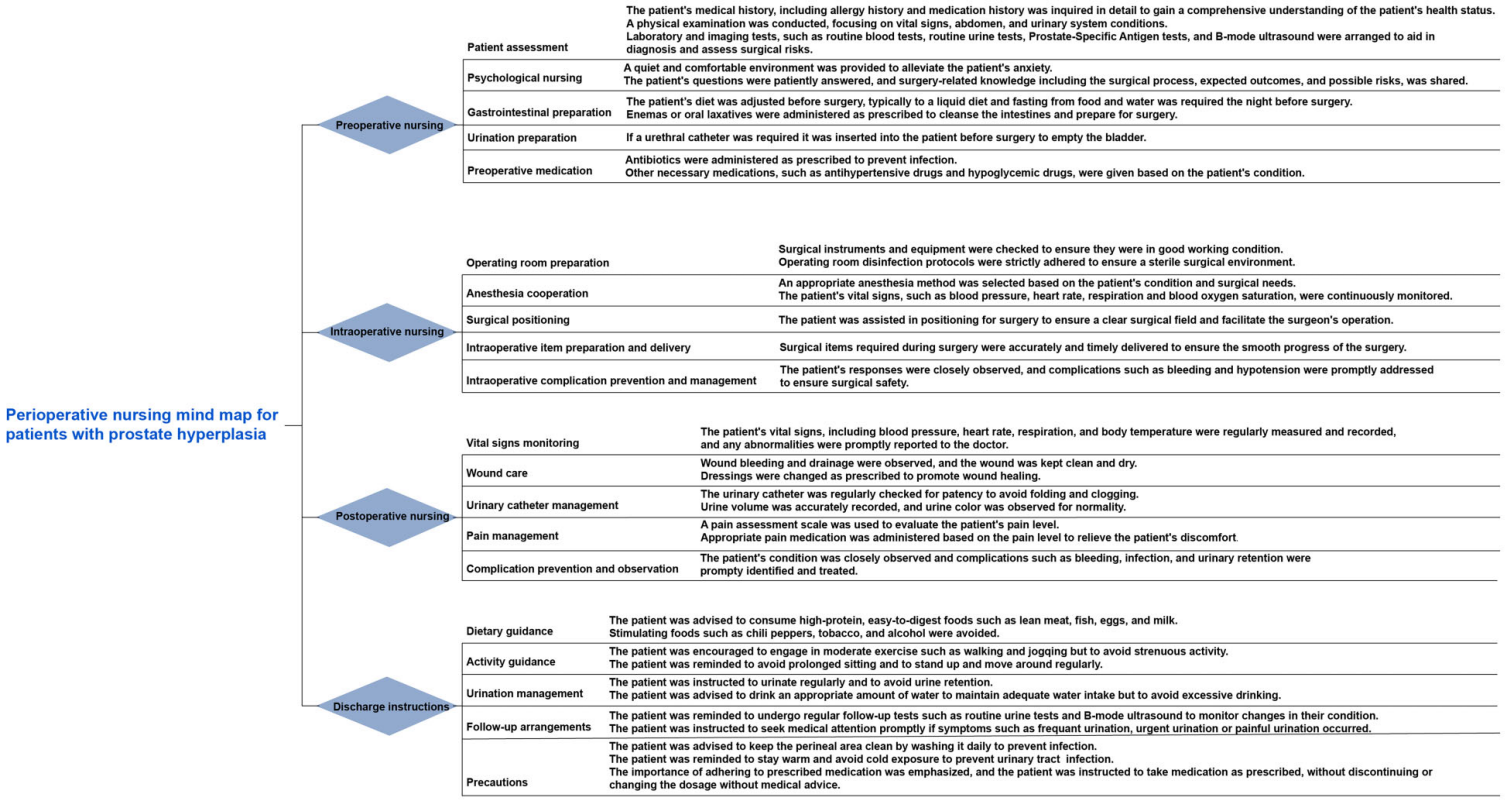
Perioperative nursing mind map for patients with prostate hyperplasia undergoing transurethral resection of the prostate.

The intervention group received mind map-guided nursing, and the specific nursing intervention methods were as follows: 1) *Establishment of the mind map nursing team*: the team comprised at least one associate senior doctor, one head nurse with more than five years of clinical nursing experience and prior leadership responsibilities, four nurses, and two doctors with more than five years of clinical practice. 2) *Development of mind maps*: using urinary incontinence and prostatic hyperplasia as keywords, a “Perioperative Nursing Mind Map for Patients with Prostatic Hyperplasia” was created based on literature review (Figure 1). This mind map consisted of four primary branches - preoperative, intraoperative, postoperative, and discharge guidance - each with corresponding secondary branches. The secondary branches of preoperative preparation included patient assessment, psychological nursing, gastrointestinal preparation, urination preparation, and preoperative medication. The secondary branches of intraoperative nursing covered operating room preparation, anesthesia cooperation, surgical positioning, intraoperative supply preparation and delivery, and prevention and management of intraoperative complications. The secondary branches of postoperative nursing comprised monitoring of vital signs, wound care, urinary catheter management, pain control, and prevention and observation of complications. The secondary branches of discharge guidance included dietary recommendations, activity guidance, urination management, follow-up arrangements, and precautions. 3) *Mind map theory training*: Nursing team members received systematic training from the associate senior doctor and head nurse. The training included effective communication with patients and families, the design and execution of individualized nursing plans, monitoring of plan adherence, and instruction in the use of assessment tools and scales. In the departmental activity room, the associate senior doctor and attending physicians alternately conducted in-depth discussions with patients and their families to obtain a comprehensive understanding of their personal, familial, and occupational backgrounds. Concurrently, the principles, applications, objectives, and utilization methods of the mind map were explained in detail to ensure that patients and families fully understood and accepted its implementation. 4) *Practical application of the mind map*: The mind map nursing plan was placed on the nursing cart at the patient's bedside. During implementation, the steps and methods of the mind map were explained in detail. Patients' conditions were observed, assessed, and analyzed according to the map to facilitate accurate identification of complications. Simulation of complication scenarios was performed, followed by rapid assessment and observation based on the mind map. Preventive and remedial measures were promptly identified and executed. Nursing staff then summarized the process, and the team discussed the plan to further enhance nursing quality. In addition, promotional materials in multiple formats - including text, images, audio, and video - were prepared and regularly shared with patients and their families through WeChat groups to improve both diversity and convenience of health information dissemination. 5) *Feedback and dynamic adjustment*: Nursing staff routinely monitored the degree of patients' plan implementation. Positive reinforcement and encouragement were given to patients with good adherence, while enhanced communication was provided to those with poor compliance. Together with patients and families, the reasons for inadequate progress were analyzed, and the nursing plan was flexibly adjusted based on the actual situation. Leveraging the divergent thinking inherent in the mind map, nurses explained preventive measures in detail and addressed patients' and families' questions in a timely manner. 6) *Emotional communication and support*: Patients and their families were encouraged to share their rehabilitation experiences and feelings, thereby establishing a supportive emotional network. Monthly recreational activities were organized to strengthen emotional connections between patients and their families, and family members were reminded to maintain frequent communication with patients to foster a positive rehabilitation environment.

### Outcome assessment

The Visual Analog Scale (VAS) (11) was utilized to assess postoperative pain at 24 and 48 h. A score of 0 indicated no pain, while a score of 10 represented the most severe pain.

The number of analgesia pump activations, time to first flatus, and length of hospital stay were compared between groups.

Quality of life was compared using the Short Form-36 (SF-36) health survey (12), which assesses eight domains: physical functioning, role limitations due to physical or emotional problems, social functioning, vitality, mental health, pain, and general health perceptions. Each domain was scored and converted to a 0-100 scale, with higher scores indicating better quality of life.

The Pittsburgh Sleep Quality Index (PSQI), Self-Rating Anxiety Scale (SAS), and Self-Rating Depression Scale (SDS) were applied to evaluate sleep quality, anxiety, and depression in the two groups before and after nursing. The PSQI (13) consists of seven components (subjective sleep quality, sleep latency, sleep efficiency, sleep disturbances, sleep duration, daytime dysfunction, and use of sleep medications), with a total score ranging from 0 to 21; higher scores indicated poorer sleep quality. For the SAS (14), patients selected options according to their experiences, and raw scores were summed and converted to standard scores; values 0**-**8, no anxiety; 9**-**21, occasional anxiety; 22**-**32, mild anxiety; 33**-**44, moderate anxiety; 45**-**60, severe anxiety. For the SDS (15), the sum of 20 items was calculated and converted to a standard score; <50 indicated normal, 50-59 mild depression, 60-69 moderate depression, and ≥70 severe depression. Lower scores indicated better states.

The International Prostate Symptom Score (IPSS) scale (16) was employed to evaluate prostate function in both groups before surgery and three months after surgery. The IPSS scale consists of seven items: frequency, urgency, incomplete emptying, nocturia, intermittency, weak stream, and straining. Each item was scored from 0 to 5, with a total score range of 0-35. Severity was categorized as mild (0-7), moderate (8-19), and severe (20-35).

Patient satisfaction was evaluated using a questionnaire developed at the hospital with 19 items covering aspects such as technical skills and communication. Each item was rated on a 5-point scale, yielding a total score of 19-95. Scores ≥77 indicated very satisfied, 58-76 satisfied, 39-57 generally satisfied, and ≤38 dissatisfied. Overall satisfaction was calculated as the sum of the “very satisfied” and “satisfied” scores.

### Sample size estimation

According to the preliminary experiment, the main observational indicators (VAS score 24 h postoperatively) were 2.65±0.86 and 3.90±1.06 in the intervention and control groups, respectively. PASS 2021 software was used for sample size estimation, and an optimized design was adopted, with an α=0.05, β=0.05, power=0.95, and assuming a 1:1 allocation ratio. The calculation indicated that at least 24 patients were required per group. Considering an anticipated dropout rate of approximately 20%, a minimum of 30 patients per group was required. Ultimately, 40 patients were enrolled in each group, for a total of 80 patients.

### Statistical analysis

Data processing was conducted using SPSS (IBM, USA) version 26.0. The normality and homogeneity of the data were tested by the Shapiro-Wilk Levene's tests. Normally distributed continuous variables are reported as means±SD, independent-sample *t*-tests were adopted for intergroup comparisons, and paired *t*-tests were employed for intragroup comparisons. Categorical variables are reported as frequencies and percentages (%), and the chi-squared test was applied. A P-value <0.05 was considered statistically significant.

## Results

### VAS score

At 48 h postoperatively, VAS scores in both groups were lower compared with those at 24 h postoperatively (P<0.05). Additionally, compared to the control group, the intervention group exhibited lower VAS scores at both 24 and 48 h postoperatively (P<0.05) (Table 1).

**Table 1:** Comparison of visual analog scale scores of the mind map-guided nursing group and the control group after transurethral resection of the prostate.

Group	24 h postoperatively	48 h postoperatively
Control group	3.90±1.06	3.55±0.88*
Intervention group	2.65±0.86	2.28±0.78*
*t* value	5.790	6.861
P value	<0.001	<0.001

Data are reported as mean±SD (n=40). *P<0.05 *vs* 24 h; independent-sample *t*-test.

### Analgesia pump use, bowel recovery, and hospitalization

After nursing, the intervention group demonstrated fewer self-administered analgesia pump activations, earlier postoperative flatus, and a shorter length of stay compared to the control group (P<0.05) (Table 2).

**Table 2:** Comparison of controlled times of analgesia pump, flatus time, and length of hospital stay of the mind map-guided nursing group and the control group after transurethral resection of the prostate.

Group	Controlled analgesia pump (times)	Flatus time (h)	Length of hospital stay (day)
Control group	10.65±1.48	31.15±2.78	12.33±1.76
Intervention group	5.90±1.19	25.18±0.59	8.60±1.60
*t* value	15.816	13.299	9.911
P value	<0.001	<0.001	<0.001

Data are reported as mean±SD (n=40); independent-sample *t*-test.

### Quality of life

After nursing, the SF-36 scores in both groups were higher compared to those before nursing (P<0.05). Furthermore, the intervention group achieved higher SF-36 scores in all dimensions compared with the control group (P<0.001) (Table 3).

**Table 3:** Comparison of Short Form-36 (SF-36) scores before and after nursing of the mind map-guided nursing group and the control group after transurethral resection of the prostate.

Group	Physical functioning		Social role		Emotional state		Vitality	
	Before	After	Before	After	Before	After	Before	After
Control group	63.95±7.03	78.23±6.66*	65.23±6.88	69.50±6.85*	60.95±7.18	80.55±6.63*	67.15±5.84	78.28±5.82*
Intervention group	63.90±6.59	90.83±5.79*	66.33±8.31	90.60±6.08*	60.85±7.08	90.65±5.81*	66.58±5.47	88.45±5.14*
*t* value	0.033	9.031	0.645	14.371	0.063	7.248	0.455	8.283
P value	0.974	<0.001	0.521	<0.001	0.95	<0.001	0.651	<0.001

Data are reported as mean±SD (n=40). *P<0.05 *vs* the same group before nursing; paired *t*-test.

### PSQI, SAS, and SDS scores

After nursing, PSQI, SAS, and SDS scores in both groups were lower compared to those before nursing (P<0.05). Moreover, reductions were more pronounced in the intervention group, whose scores were significantly lower than those of the control group (P<0.05) (Table 4).

**Table 4:** Comparison of PSQI, SAS, and SDS scores of the mind map-guided nursing group and the control group after transurethral resection of the prostate.

Group	PSQI score		SAS score		SDS score	
	Before nursing	After nursing	Before nursing	After nursing	Before nursing	After nursing
Control group	14.18±1.30	7.68±1.05*	43.88±5.44	31.33±7.41*	58.35±3.74	43.95±3.52*
Intervention group	14.13±1.24	4.80±1.26*	43.58±3.73	23.35±5.86*	58.50±3.18	35.68±2.75*
*t* value	0.176	11.072	0.288	5.341	0.193	11.728
P value	0.861	<0.001	0.774	<0.001	0.847	<0.001

Data are reported as mean±SD (n=40). *P<0.05 *vs* the same group before nursing. PSQI: Pittsburgh Sleep Quality Index; SAS: Self-Rating Anxiety Scale; SDS: Self-Rating Depression Scale.

### IPSS scale

Baseline IPSS scores did not differ significantly between groups (P>0.05). After nursing, the intervention group had lower IPSS scores compared to the control group (P<0.05) (Table 5).

**Table 5:** Comparison of IPSS scores of the mind map-guided nursing group and the control group after transurethral resection of the prostate.

Group	IPSS score	
	Before nursing	After nursing
Control group	25.68±2.87	8.33±2.32*
Intervention group	25.40±2.81	5.78±1.97*
*t* value	0.433	5.295
P value	0.666	<0.001

Data are reported as mean±SD (n=40). *P<0.05 *vs* the same group before nursing; paired *t*-test. IPSS: International Prostate Symptom Score.

### Nursing satisfaction

After nursing, the intervention group exhibited a higher nursing satisfaction rate compared to the control group (P<0.05) (Table 6).

**Table 6:** Comparison of nursing satisfaction between the mind map-guided nursing group and the control group after transurethral resection of the prostate.

Group	Very satisfied	Satisfied	Generally satisfied	Dissatisfied	Overall satisfaction
Control group	10 (25%)	17 (42%)	9 (22.5%)	4 (10%)	27 (67.5%)
Intervention group	20 (50%)	18 (45%)	2 (5%)	0 (0%)	38 (95%)
X^2^ value	9.928				
P value	0.002				

Data are reported as number and percentage of n=40 per group.

## Discussion

BPH is a primary cause of lower urinary tract symptoms, including difficult urination, nocturia, urinary urgency and frequency, bladder activation or weak urine stream, and difficulty in emptying. TURP remains the gold standard surgical treatment for BPH, as it effectively relieves bladder outlet obstruction and reduces catheter dependence (17). Nevertheless, postoperative pain is common after TURP and may hinder recovery. The present study therefore explored the effect of mind map-guided nursing on postoperative pain control in this population.

Mind maps, originally developed as an educational tool, have been widely applied in medical training, including in anatomy, community medicine, physical therapy, and chiropractic education. By presenting information in a visual and non-linear format, mind maps foster visuospatial associations and facilitate deeper understanding, thereby enhancing information organization and retention (7). Beyond education, mind maps have also been increasingly introduced into clinical nursing practice. For example, studies have shown that mind map-based comprehensive nursing interventions can reduce anxiety and depression in breast cancer patients, alleviate physiological stress and fatigue, and improve sleep quality (18). Similarly, Wang et al. (19) demonstrated that perioperative nursing supported by 3D printing combined with mind mapping can improve psychological well-being, enhance quality of life, and reduce complication rates. In patients with severe brain injury, a goal-oriented nursing model structured by mind maps has been shown to accelerate hospital discharge, decrease complication incidence, and promote neurological and motor function recovery (20). Furthermore, Ma et al. (21) reported that a multidisciplinary nursing model integrated with mind map teaching improved postoperative care for advanced pancreatic cancer patients by alleviating negative emotions, enhancing quality of life, reducing pain, lowering complication rates, and increasing nursing satisfaction. Collectively, these findings underscore the potential of mind map-guided approaches to improve nursing quality and patient outcomes.

This study further confirmed the effectiveness of mind map-guided nursing in alleviating postoperative pain. Specifically, we observed that the VAS scores of the intervention group at 24 and 48 h after surgery were significantly lower than those of the control group, indicating that mind map-guided nursing can effectively relieve pain following TURP. In addition, compared with the control group, the intervention group demonstrated fewer analgesic pump activations, shorter time to first flatus, and reduced length of hospital stay. These findings suggest that mind map-guided nursing optimizes pain management, decreases reliance on analgesic medications, promotes postoperative recovery, and shortens hospitalization. Furthermore, the intervention group achieved higher SF-36 scores across all dimensions and lower PSQI, SAS, and SDS scores compared with the control group, reflecting improvements in quality of life and mental health. Moreover, the intervention group exhibited lower IPSS scores than the control group, indicating that mind map-guided nursing contributed to alleviating prostate-related symptoms and enhancing treatment outcomes. Nursing satisfaction was also higher in the intervention group, suggesting that patients were more accepting of and satisfied with this mind map-based nursing model.

Previous studies provide additional evidence supporting structured nursing models after TURP. For example, continuous psychological nursing based on the grey clustering algorithm, combined with pelvic floor muscle exercises, has been shown to significantly improve postoperative erectile function, reduce complications, and alleviate anxiety and depression (22). Cluster nursing based on the 10S CQI has been reported to accelerate recovery in BPH patients undergoing TURP, improve psychological state and quality of life, and reduce the incidence of delirium and other complications (23). Likewise, continuity of care based on the theory of behavior change has demonstrated favorable outcomes in patients with overactive bladder following TURP, improving urinary status, enhancing self-care ability and quality of life, and increasing nursing satisfaction (24).

When compared with these evidence-based nursing practices, the unique value and potential advantage of mind map-guided nursing lie in its provision of a highly structured and visual methodological tool, rather than a single content-based intervention. For instance, psychological nursing based on the grey clustering algorithm or cluster nursing based on the 10S CQI establish specific nursing frameworks, such as psychological support or quality improvement processes. In contrast, mind mapping allows these complex processes to be visually integrated and presented in an intuitive and systematic manner. With postoperative recovery as the central theme, the mind map radiates outward to cover diverse nursing components, such as patient assessment, psychological counseling, pain management, and discharge planning, thereby ensuring comprehensive care and organized implementation. This systematic and visualized presentation not only helps standardize nursing behaviors, minimize omissions, and achieve consistency in nursing practice but also transforms complex rehabilitation protocols into graphical information, enhancing patients' comprehension and adherence. This may explain the superior effects of mind map-guided nursing compared with conventional nursing, particularly in terms of pain relief, psychological state improvement (SAS/SDS), sleep quality (PSQI), and nursing satisfaction. Therefore, as an integrative and implementation-oriented tool, mind map-guided nursing has the potential to incorporate the essence of multiple evidence-based practices and translate them efficiently into clinical application, thereby demonstrating unique advantages for clinical promotion.

Nevertheless, this study has certain limitations. First, the sample size was relatively small and may not adequately represent the broader population of TURP patients. Second, the absence of long-term follow-up data limits our ability to comprehensively evaluate the sustained impact of mind map-guided nursing throughout the recovery process. Third, the lack of qualitative methodologies restricts our understanding of patients' subjective experiences and perceptions regarding the intervention.

In summary, this study confirmed that the implementation of mind map-guided nursing holds important clinical value in controlling postoperative pain after TURP. It not only reduced the frequency of analgesic pump use, shortened time to flatus and length of hospital stay, and improved patients' quality of life, but also enhanced sleep quality and psychological well-being, promoted prostate function recovery, and increased nursing satisfaction. Therefore, this nursing model warrants further clinical promotion and application. Given the limitations of this study, future research should include larger sample sizes and more diverse patient populations to enhance the generalizability of findings. In addition, long-term follow-up studies are needed to continuously monitor pain, recovery trajectories, and changes in quality of life after implementing mind map-guided nursing. Furthermore, qualitative methods such as interviews or focus groups could provide deeper insights into patients' perceptions of the intervention's effectiveness and acceptability. Future work may also explore the application of mind map-guided nursing in other aspects of post-TURP care, as well as its potential extension to other surgical and disease management contexts. Comparative studies with other evidence-based nursing interventions are also needed to clarify the relative strengths and limitations of mind map-guided nursing, thereby promoting its continuous refinement and advancement in nursing practice.

## Data Availability

All data generated or analyzed during this study are included in this published article.
